# Prevalence of maternal preconception risk factors: an Italian multicenter survey

**DOI:** 10.1186/s13052-014-0091-5

**Published:** 2014-11-23

**Authors:** Pierpaolo Mastroiacovo, Roy Miodini Nilsen, Emanuele Leoncini, Paolo Gastaldi, Valentina Allegri, Arianna Boiani, Francesca Faravelli, Federica Ferrazzoli, Andrea Guala, Valeria Madrigali, Gioacchino Scarano

**Affiliations:** Alessandra Lisi International Centre on Birth Defects and Prematurity, Via Carlo Mirabello 14, Rome, 00195 Italy; Department of Global Public Health and Primary Care, University of Bergen, Bergen, Norway; Unit (UOC) Obstetrics and Gynecology, Santo Spirito in Saxia Hospital, Rome, Italy; Unit (U.O.) of Pediatrics, Vaio Hospital, Fidenza, Parma, Italy; Department of Mother and Child health, S. Giovanni Calibita-Fatebenefratelli Hospital, Rome, Italy; Medical Genetics Unit, Galliera Hospital, Genoa, Italy; Unit of Neonatology, Santo Spirito in Saxia Hospital, Rome, Italy; Unit of Pediatrics, Castelli Hospital, Verbania, Italy; Unit of Neonatology and Neonatal Intensive Care, Azienda Ospedaliero-Univesitaria Pisana, Pisa, Italy; Department of Medical Genetics, Gaetano Rummo Hospital, Benevento, Italy

**Keywords:** Italy, Preconception, Pregnancy, Prevalence, Risk factors

## Abstract

**Objectives:**

Adequate preconception maternal health care is essential to reduce the risk of unwanted pregnancy outcomes and complications. Still, many women are exposed to a number of unhealthy risk factors both before and early in pregnancy. This study aimed to estimate the prevalence of a number of important preconception risk factors using data from a recent multicenter study in Italy.

**Methods:**

The study was based on cross-sectional data from seven maternity clinics located in six different regions in Italy during the period January – June, 2012. Data on maternal preconception risk factors and characteristics were collected from 1,892 women who delivered healthy children and 320 women who were pregnant in the first trimester.

**Results:**

About 97% of the women (n = 2,212) were exposed to one or more preconception risk factors. The overall prevalence of the most essential maternal risk factors was as follows: 41% had a age ≥35 years, 36% mistimed or did not intend their pregnancy, 58% did not request a preconception health visit to their doctor, 76% did not use folic acid supplements before pregnancy, 26% smoked at the last menstrual period, 19% had a body mass index ≥25 kg/m^2^ before pregnancy, and 10% suffered from pregestational chronic diseases. The prevalence of certain variables varied between the maternity clinics.

**Conclusions:**

Many Italian women are exposed to a number of preconception risk factors that have been associated with adverse pregnancy complications and outcomes. More effective intervention programs to improve preconception health in Italian women are strongly needed.

## Introduction

Embryonic development in humans occurs from the time of conception until the end of 10^th^ from the last menstrual period (the first 56 days - eight completed weeks of embryonic development) [1]. Environmental and genetic interference *in-utero* during this early time period, especially soon after conception and during the first five weeks of embryonic development, could be harmful for the developing embryo, resulting in severe adverse reproductive outcomes including congenital malformations [2]. Consequently, to reduce the risk of such unwanted pregnancy outcomes, good maternal health and health awareness should be obtained already before the onset of pregnancy or early as possible during pregnancy.

In 2006, Centers for Disease Control and Prevention published 10 recommendations to improve preconception and inter-conception health care for women before pregnancy [3]. It defined a set of interventions to identify and modify biomedical, behavioral, and social risks to a woman's health or pregnancy outcome through prevention and management. However, in order to publish such public health recommendations, it is essential to have sufficient knowledge of the prevalence and patterns of preconception risk factors. Developing specific monitoring systems that track maternal behaviors, experiences, and health conditions, including preconception health, as done in the United States, would be beneficial [4].

In Italy, a large portion of women smoke before pregnancy, do not take folic acid supplements as recommended, and have an advanced age when pregnant for the first time [5-9], but information about the prevalence of other maternal health-related characteristics or behaviors before the onset of a pregnancy is limited [10]. Additionally, there are no central data bases to retrieve preconception data for estimating representative prevalences on the national level. In this study, we therefore conducted a multicenter survey among women giving birth in Italy with the primary aim to assess the prevalences of a wide range of established preconception risk factors for adverse pregnancy outcomes and complication.

## Methods

### Study population

The present study was a cross-sectional survey conducted between January and June 2012, in seven Italian maternity clinics located in six different regions (three in the North Italy, three in the Central Italy and one in the South Italy): Castelli Hospital (Verbania, Piemonte), Vaio Hospital (Fidenza, Emilia-Romagna), Galliera Hospital (Genova, Liguria), Santa Chiara Hospital (Pisa, Toscana), Fatebenefratelli Hospital and Santo Spirito Hospital (Roma, Lazio), Rummo Hospital (Benevento, Campania). All maternity clinics are located in major cities of Italy, with the number of inhabitants varying from 25,00 in Fidenza to 88,000 in Pisa up to 600,000 in Genova and 2.8 milion in Rome. The gross domestic product (GPD) per capita is similar in the north and centrat Italy regions, ranging between 21,000 Euro/year in Liguria and 24,000 Euro/year in Emilia Romagna, while in Campania the GPD per capita is 12, 800. The study was approved by the Ethical Committees for each of the participating maternity clinics.

The study included convenience sample of pregnant women at their first prenatal visit (n = 320, Verbania only) and mothers of recently delivered healthy newborns (n = 1,892; the other six participating maternity clinics). Mothers of newborns with any congenital malformation, with low birth weight (<2500 g), preterm birth (<37 weeks), or with children admitted to the neonatal intensive care were not invited to participate to avoid subjecting them to unnecessary stress. In Verbania, however, women were invited during early pregnancy, and therefore no such exclusions were done. Also women not speaking Italian were excluded from the present study. All women were consecutively included in the study during the study period.

Among the seven participating maternity clinics, a total of 2,212 women were recruited between January and June 2012 (Table 1). The largest proportion of participants was from Roma Fatebenefratelli (23.1%) while the lowest was from Pisa (9.1%). The percentage of participating women as a fraction of invited was approximately 100% at each maternity clinic. None of the participating women withdrew during the study and all participants completed the questionnaire.

**Table 1:** **Distribution of mothers according to maternity clinics and region**

		**Study population**	
**Maternity clinics**	**Region**	**n**	**%**
All		2 212	100
Verbania	Piemonte	320	14.5
Genova	Liguria	393	17.8
Fidenza	Emilia-Romagna	220	9.9
Pisa	Toscana	202	9.1
Roma Fatebenefratelli	Lazio	511	23.1
Roma Santo Spirito	Lazio	240	10.8
Benevento	Campania	326	14.7

### Data collection

All women were asked to fill in a self-administered questionnaire without identities (names, address, birth date) at their first prenatal visit (in Verbania the average was at 6.8 weeks of gestation) or before discharge from the maternity clinic (in the other six participating maternity clinics the average was 3.0 days after the delivery). The following topics were covered (the questionnaire can be provided on request to the corresponding author): (a) several questions on previous and present pregnancy history (e.g. date of last menstrual period (LMP), date of delivery, previous pregnancy outcomes), (b) three questions on pregnancy intention, (c) three question on preconception health visit and infertility treatment, (d) nine questions on protective factors (e.g. folic acid supplementation) and risk factors (e.g. smoking, medications, pregestational chronic diseases) in pregnancy, and (e) eight questions on health-related characteristics (e.g., age, marital status, educational level). The answers to the questions were mainly organized in categories or multiple choices. Upon returning the questionnaire, the answers were reviewed by the attending doctor. If data were missing, the women were asked to fill in missing answers unless she did not remember or was not able to answer. The questionnaire’s comprehensiveness and feasibility was evaluated as adequate in a preliminary examination of 50 women prior to the current study (unpublished).

In the present study, the term “preconception” or “before pregnancy” was defined as the time period before the first day at the LMP. A pregnancy was defined intended, mistimed or unintended on the basis of the score of the intensity of pregnancy planning effort developed by Morin et al. [11], intended score: 9–12, mistimed score: 4–8 and unintended score: 0–3. The preconception health visit was defined as the specific request of the woman to her health care provider in the year before pregnancy to ask for advice on a near future pregnancy and more specifically what medical examinations to perform, medications and vitamins to take.

Use of medications during the first trimester was defined as the use of any prescribed or over-the-counter medication for at least two consecutive days or for at least three times in a week. Second-hand smoking was defined as exposure for at least one hour per day passed in a room, at home, or in a car or in office, with one or more smokers. Body mass index (BMI) before pregnancy was calculated as the body weight divided by the height squared (kg/m^2^) and categorized into four groups according to the World Health Organization criteria: underweight (<18.5), normal weight (18.5–24.9), overweight (25–29.9), obese (≥30 kg/m^2^) [12].

### Statistical analysis

**Figure 1:**
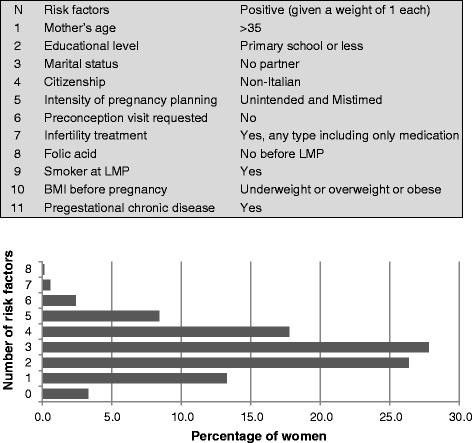
**Distribution of mothers according to the number of preconception risk factors.** Upper panel defines the weights of 11 risk factors, while the lower panel shows the distribution of mothers according to the number of preconception risk factors.

We estimated the prevalence of each maternal characteristic and preconception risk factor overall as well as for each participating maternity clinic. The prevalence for each variable was estimated as a fraction of non-missing values. Missing values were less than 3% for all variables analyzed except for passive smoking exposure (9%) and medication use (5%). Because women may be exposed to more than one risk factor, they may also have an excess risk of adverse pregnancy outcomes and complications. Therefore, it is of interest to know the distribution of number of risk factors in the pregnant population. We calculated the total number of risk factors for each individual from 11 (of 14) relevant maternal characteristics and preconception risk factors variables (upper panel of Figure 1). Overall comparisons of prevalences of risk factors between maternity clinics were tested by the chi-square test of homogeneity. Notably, we detected statistically significant differences (P values less than 0.05) in all risk factors, except for the prevalence of smoking at the LMP.

## Results

### Maternal characteristics

Of the 2,212 women included in the present survey, the mean maternal age at delivery was 33 (range 13–50) years, 6.1% were younger than 25 years while 40.9% were older than 35 years; 40.7% had university degree as the highest educational level and 15.4% primary school; 90.2% were married or cohabiting, and 14.5% was of foreign origin; 81.9% had a full or part time work outside home (Table 2). Roma Fatebenefratelli had the highest proportion of women giving birth at ≥35 years (48.8%) and the highest proportion of women with a university degree (56.8%); Verbania and Benevento had the lowest proportion of women with a university degree (25.7% and 29.8%, respectively), and the lowest proportion of non-Italian citizens (6.9% and 5.2%, respectively).

**Table 2:** **Maternal characteristic according to maternity clinics**

**Background variables**	**All (n = 2 212)**		**Verbania (n = 320)**	**Genova (n = 393)**	**Fidenza (n = 220)**	**Pisa (n = 202)**	**Roma Fatebenefratelli (n = 511)**	**Roma Santo Spirito (n = 240)**	**Benevento (n = 326)**
	**n**	**%**	**%**	**%**	**%**	**%**	**%**	**%**	**%**
Mother’s age									
<25	131	6.1	7.5	7.6	6.0	2.5	2.6	8.1	9.5
25-29	405	18.9	21.9	18.8	21.5	13.9	12.9	25.0	22.3
30-34	733	34.1	34.2	32.1	36.0	35.3	35.7	31.4	34.1
35-39	669	31.1	28.2	31.8	28.5	35.3	37.3	25.4	26.4
40 or more	211	9.8	8.2	9.7	8.0	12.9	11.5	10.2	7.8
Educational level									
Primary school	340	15.4	28.5	14.0	18.4	9.5	4.5	12.5	25.2
Secondary school	966	43.9	45.8	43.5	45.2	45.0	38.7	49.2	45.1
University	896	40.7	25.7	42.5	36.4	45.5	56.8	38.3	29.8
Marital status									
Married or living together	1 974	90.2	91.8	83.5	90.2	92.5	87.6	99.2	92.6
No partner	215	9.8	8.2	16.5	9.8	7.5	12.4	0.8	7.4
Citizenship									
Italian	1 887	85.5	93.1	75.1	85.8	89.1	88.8	69.2	94.8
Non-Italian	321	14.5	6.9	24.9	14.2	10.9	11.2	30.8	5.2
Working activity									
Studying	107	4.9	2.8	3.6	2.3	7.0	5.9	7.9	5.3
Working	1 788	81.9	87.5	80.4	88.5	87.6	87.3	83.3	60.7
Working at home (housewife)	288	13.2	9.7	16.0	9.2	5.5	6.9	8.8	34.0

### Present pregnancy history

Nearly two-thirds of women reported that they had planned their pregnancy (64.5%), while 35.5% reported that they had mistimed or did not plan their pregnancy (Table 3). A preconception health visit to a doctor was requested by only 41.5% of women (among those who intended their pregnancy 55.4%, among those who mistimed their pregnancy 20%, among those who did not intend their pregnancy 0%). Infertility treatment was reported by 7.5% of women (1.4% only use of ovulation inductors). The highest proportion of women who planned their pregnancy was observed in Genova (76.3%), the lowest in Benevento (52.0%). The highest proportion of women who requested preconception health visit to their health care provider was observed in Roma Fatebenefratelli (58.7%), while the lowest in Benevento (13.2%). When requested, the preconception health visit was performed more often by a gynecologist (90.3%) and sometimes by the general practitioner (9.7%).

**Table 3:** **Present pregnancy history according to maternity clinics**

**Present pregnancy history**	**All (n = 2 212)**		**Verbania (n = 320)**	**Genova (n = 393)**	**Fidenza (n = 220)**	**Pisa (n = 202)**	**Roma Fatebenefratelli (n = 511)**	**Roma Santo Spirito (n = 240)**	**Benevento (n = 326)**
	**n**	**%**	**%**	**%**	**%**	**%**	**%**	**%**	**%**
Pregnancy intention									
Unintended	121	5.6	6.1	6.4	3.2	4.0	4.5	7.6	6.9
Mistimed	652	29.9	33.4	17.3	31.1	33.7	32.9	21.1	41.2
Intended	1 406	64.5	60.5	76.3	65.8	62.3	62.6	71.3	52.0
Preconception health visit									
Yes	911	41.5	46.1	40.2	37.2	39.1	58.7	45.0	13.2
No	1 283	58.5	53.9	59.8	62.8	60.9	41.3	55.0	86.8
Infertility treatment									
No	2 017	92.4	96.2	93.4	89.6	89.9	87.8	93.7	97.5
Ovulation inductor use, no ART	31	1.4	0.0	2.8	0.9	2.5	1.2	2.5	0.3
ICSI. GIFT	66	3.0	2.5	2.8	4.7	2.0	5.5	1.3	0.6
FIVET. IUI	64	2.9	1.3	1.0	3.8	5.0	5.5	2.1	1.6
ART. not specified	4	0.2	0.0	0.0	0.9	0.5	0.0	0.4	0.0

### Preconception risk factors

#### Maternal folic acid use

Of the participating women (women with missing information on folic acid use were excluded, n = 23), 1,838 (84.0%) reported having used folic acid supplements in connection with pregnancy: 11.8% had initiated use six months before pregnancy; 11.7% had initiated use one month before pregnancy, giving a total prevalence of use before pregnancy of 23.5%; 54.9% of women had initiated use within the first trimester. A small portion reported starting use after the first trimester (5.6%) (Table 4). The lowest preconception folic acid supplement use (at least one month before pregnancy) was observed among women giving birth in the Benevento (11.2%) and in Fidenza (16.6%), while the highest was observed at the maternity clinics in Roma Santo Spirito and Genova (31.7% and 31.0%, respectively).

**Table 4:** **Preconception risk factors according to maternity clinics**

**Preconception risk factors**	**All (n = 2 212)**		**Verbania (n = 320)**	**Genova (n = 393)**	**Fidenza (n = 220)**	**Pisa (n = 202)**	**Roma Fatebenefratelli (n = 511)**	**Roma Santo Spirito (n = 240)**	**Benevento (n = 326)**
	**n**	**%**	**%**	**%**	**%**	**%**	**%**	**%**	**%**
Folic acid									
No	351	16.0	39.9	11.7	13.4	14.5	10.8	9.6	13.7
At least one month before	256	11.7	7.9	15.0	8.8	11.0	14.3	16.3	6.2
At least six month before	259	11.8	8.2	16.0	7.8	14.5	14.1	15.4	5.0
Soon after pregnancy diagnosis	1201	54.9	40.2	53.7	59.0	55.5	53.8	57.5	67.3
After 1 trimester	122	5.6	3.8	3.6	11.1	4.5	7.0	1.3	7.8
Smoking at LMP									
Yes	583	26.4	23.1	22.6	27.3	32.7	25.8	28.3	28.5
No	1 629	73.6	76.9	77.4	72.3	67.3	74.2	71.7	71.5
Smoking after pregnancy confirmation									
Yes	181	8.2	7.2	8.4	10.5	9.4	7.0	7.1	9.2
No	2 031	91.8	92.8	91.6	89.5	90.6	93.0	92.9	90.8
Second-hand smoking									
Yes	287	14.3	6.1	10.9	11.5	9.7	17.0	11.2	27.8
No	1 715	85.7	93.9	89.1	88.5	90.3	83.0	88.8	72.2
BMI before pregnancy									
Underweight	184	8.4	9.7	11.7	6.5	7.5	7.9	9.2	4.9
Normal range	1 588	72.2	71.1	71.8	75.6	72.1	78.3	70.4	63.4
Overweight	305	13.9	14.2	12.0	13.8	15.9	10.7	12.5	20.6
Obese	123	5.6	5.0	4.6	4.1	4.5	3.2	7.9	11.1
Pregestational disease									
Yes	208	9.5	6.4	7.4	6.9	12.9	16.6	6.9	5.6
No	1 981	90.5	93.6	92.6	93.1	87.1	83.4	93.1	94.4
Specific disease									
Hypothyroidism	117	5.3	3.2	2.5	5.0	7.5	9.7	5.2	3.1
Thrombophilia	16	0.7	0.3	1.0	0.5	0.5	1.6	0.0	0.3
Hypertension	13	0.6	0.6	0.5	0.0	0.5	1.4	0.0	0.3
Asthma	10	0.5	0.3	0.5	0.0	0.5	0.6	0.4	0.6
Medication use during first trimester									
Yes	539	25.7	17.7	24.3	27.6	28.2	36.0	16.4	23.7
No	1 562	74.3	82.3	75.7	72.4	71.8	64.0	83.6	76.3

#### Maternal prenatal smoking

Reported smoking prevalence at the LMP was overall 26.4% (not statistically significant different among the seven participating health clinics, range: 22.6% in Genova and 32.7% in Pisa) (Table 4). Among smokers, 69.0% reported that they had quit smoking at the time of pregnancy confirmation, yielding an overall smoking prevalence during pregnancy after pregnancy confirmation of 8.2%. Participating women from Roma Santo Spirito were more often quitters than others (75.3%), followed by Roma Fatebenefratelli (73.2%) and Pisa (71.1%). The overall prevalence of second-hand smoking varied widely between women from different maternity clinics: from as low as 6.1% in Verbania to as high as 27.8% in Benevento.

#### Maternal morbidity and medication use

The overall reported prevalence of underweight, overweight and obesity before pregnancy was 8.4%, 13.9% and 5.6% respectively (Table 4). Compared to the overall population, Roma Fatebenefratelli had the lowest proportion of obese (3.2%) while the highest (11.1%) was observed at Benevento.

The reported prevalence of pregestational chronic diseases before pregnancy in the total group of women was 9.5%: hypothyroidism (5.3%), thrombophilia (0.7%), hypertension (0.6%) and asthma (0.5%) were the more frequent conditions. Diabetes mellitus was reported in 0.4% of the participants. Medication use during first trimester was reported by 539 (25.7%) women. Overall, use of paracetamol (7.2%), eutirox (5.3%), and antibiotics (5.0%) were the most frequently used medications (Table 4).

### Multiple preconception risk factors

The overall number of preconception risk factors varied from 0 to 8 (lower panel of Figure 1), with a median at 3 (interquartile range 2–4). Furthermore, 3.3% of the mothers did not report any risk factor, 13.3% reported one risk factor, while 83.5% of women were exposed to two or more risk factors before their pregnancy.

## Discussion

This is the first study to assess the prevalence of a number of maternal preconception risk factors in mothers giving birth in Italy. About 97% of the women were exposed to one or more preconception risk factors. The overall prevalence of the most essential risk factors was as follows: maternal age ≥35 years: 41%, unintended or misted pregnancy: 36%, preconception health visit not requested: 58%, maternal folic acid supplement not used before pregnancy: 76%, maternal smoking at LMP: 26%, maternal BMI ≥25 kg/m^2^ before pregnancy: 19%, maternal chronic diseases: 10%. The prevalence of certain variables varied somewhat between maternity clinics, possibly due to regional differences in socioeconomic levels and lifestyle habits. Particularly, the maternity clinic in Benevento, which is located in the South of Italy, had the lowest portion of women with higher education and the highest proportions of housewives.

This was a large multicenter study with a standardized data collection from seven maternity clinics (three in the north, three in the center, and one in the south). Additionally, it comprised a large number of preconception risk factors, of which some, to our knowledge, has not previously been reported in Italy before: intensity of pregnancy planning effort, preconception health visit, and second hand smoking. Consequently, our study results provide an important basis for further work on preconception maternal health care in Italian women and their offspring. However, estimates should be interpreted with some caution. First, maternity clinics may not have been representative of their respective region. Further, all participating women had delivered live born babies with birth weights of 2500 g or more and they had no significant complications during their pregnancy. Estimates may, therefore, not be entirely generalizable to all pregnant women.

Advanced maternal age is of major concern in reproductive epidemiology and is associated with Down’s syndrome as well as other pregnancy outcomes and complications [13]. In the present study, the overall prevalence of maternal age ≥35 years was 41%, which is slightly higher than that reported by the National Institute for Statistics (33%) in Italy in 2012 [14]. Compared to other countries in Europe, Italy has one of the highest prevalence of women delivering at 35 years or more [9]. To understand why a large portion of Italian women have their first pregnancy late in life, merging the field of medical and social sciences would be interesting.

To our knowledge, valid data on pregnancy intention are not available in Italy. Using a standardized tool to evaluate pregnancy intention [11], we found that 64.5% of the participating women reported that they had intended their pregnancy. Since one of three infants is born from a pregnancy that was mistimed or not intended and since such pregnancies are linked to poor pregnancy outcome [15], public health actions are strongly recommended to increase the number of planned pregnancies. Pregnancy planning increases health awareness and help women modify unhealthy lifestyle habits before entering a pregnancy. Also adequate contraception education and use may help reduce the large number of unintended and mistimed pregnancies.

Folic acid is a synthetic B-vitamin found in dietary supplements, recommended to fertile women to lower the risk of having a baby with neural tube defects [16]. In Italy, the official recommendations from April 2004 state that all women planning a pregnancy, and those who do not actively exclude the possibility of becoming pregnant, should take at least 0.4 mg per day of folic acid from at least one month before pregnancy. In the present study, preconception use of folic acid supplements before pregnancy was only 24.0%, a prevalence that is consistent with what is reported in an a representative sample of Italian women (i.e., 20.8% in 2008 and 25.4% in 2010) [10]. The use of supplementation in Italy is increasing, but still lower than that reported in other countries [17,18], indicating that more efforts are needed to increase the use of this B vitamin before and early in pregnancy in Italian women.

Maternal smoking before pregnancy and during the first week of pregnancy is associated with infertility [19], subfertility [20], as well as birth defects [21]. In this study, we found that the smoking prevalence was 26.4% at the LMP and 8.2% after pregnancy recognition. These prevalences are slightly higher than that reported in a previous Italian population-based study conducted in 2009; 21.6% before and 6.7% during pregnancy [6]. These estimates remain far from the goal of 1.4% of smoking prevalence during pregnancy given for example by “Healthy People – 2020” in United States where the prevalence of smoking during pregnancy was 10.4% in 2007 [22].

Overweight and obesity is an increasing health problem in the world. Obesity is associated with many adverse reproductive outcomes including subfertility [23], spontaneous abortions [24], stillbirths [25], and birth defects [26]. In this study, the prevalence of overweight and obesity was estimated to be 13.9% and 5.6% respectively and is lower than that observed in other European countries [9]. In a survey conducted in 2011, the prevalence of overweight and obesity in an Italian population of 18–34 years of age was 20% for men and 4% for women [27].

In Scandinavia, nationwide birth registries have been implemented since the 1960s with the primary aim to monitoring congenital malformation and unwanted pregnancy outcomes and complications [28,29]. Also information on preconception folic acid use, maternal smoking before and during pregnancy, as well as obesity has been included in these registrations systems [30,31]. In United States, the well-developed monitoring system Pregnancy Risk Assessment Monitoring System [4], collects data regarding maternal behaviors, experiences, and health before, during, and after pregnancy, and give estimates of several preconception health characteristics and it is a useful system to derive preventive policies. In Italy, some limited birth data are collected through the CeDAP (certificate of delivery care). This national registration system could be enhanced by using a self-administered questionnaire (like that in the present study) on most important preconception risk factors to obtain a nationwide standardized preconception surveillance system. Information from such a system would help health care workers to better plan intervention programs designed for improving maternal health.

In our study, 97% of the participating women reported one or more risk factors. Although, the set of chosen risk factors were study specific, a similar estimate (98%) was calculated in a recent study of couples contemplating pregnancy [32]. Both these studies included risk factors on unhealthy lifestyle, and indicate that promoting preconception health is an essential component of any broad strategy to prevent adverse reproductive outcomes.

Using data from a multicenter study in Italy, we found that many Italian women were exposed to a number of preconception risk factors that have previously been associated with adverse pregnancy complications and outcomes. Some of these risk factors are increasing in prevalence and it is now time to put more efforts in reversing these trends. In order to achieve more knowledge on preconception risk factors and their consequences, a national preconception surveillance system, similar to those in other countries, should be implemented in Italy. A closer surveillance would help prevent a number of adverse pregnancy complications and outcomes, reduce the related costs, and increase the number of healthy children.
